# Is obesity more likely among children sharing a household with an older child with obesity?

**DOI:** 10.23889/ijpds.v8i2.2203

**Published:** 2023-09-14

**Authors:** Nicola Firman, Marta Wilk, Milena Marszalek, Lucy Griffiths, Gill Harper, Carol Dezateux

**Affiliations:** 1 Centre for Primary Care, Wolfson Institute of Population Health, Faculty of Medicine and Dentistry, Queen Mary University of London, London, United Kingdom; 2 Swansea University Medical School, Faculty of Medicine, Health & Life Sciences, Swansea, United Kingdom

## Objectives

We used a dynamic method of identifying household members from Electronic Health Records (EHRs) linked to National Child Measurement Programme (NCMP) data to estimate the likelihood of children with obesity sharing a household with an older child with obesity, accounting for individual and household characteristics.

## Method

We included 126,829 NCMP participants in four London boroughs and assigned households from encrypted Unique Property reference Numbers (UPRNs) at NCMP date for 115,466 (91%). We categorised the ethnic-adjusted body mass index of the youngest and oldest household child (underweight/healthy weight<91st, ≥91st to <98th overweight, obesity≥98th centile) and explored associations of the youngest child’s weight status with: oldest child’s weight status, number of household children (two, three or ≥4), youngest child’s sex, ethnicity and school year of NCMP participation (reception or year 6). We estimated adjusted odds ratios (aOR) and 95% confidence intervals (CI) of obesity in the youngest child.

## Results

19,702 UPRNs were shared by two or more NCMP participants (youngest children: 51.2% male, 69.5% reception). 10.4% of youngest (95% CI: 10.0,10.9) and 13.0% of oldest (12.5,14.3) children were living with obesity. One third of youngest children with obesity shared a household with another child with obesity (33.2%; 31.2,35.2), compared with 9.2% (8.8,9.7) of those with a healthy weight. Youngest children living with an older child with overweight (aOR: 2.33; 95% CI: 2.06,2.64) or obesity (4.59, 4.10,5.14), those from South Asian ethnic backgrounds (1.89; 1.64,2.19) or taking part in NCMP in year 6 (2.21; 2.00,2.43) were more likely, and girls (0.73; 0.67,0.81), children living with just one other child (0.87; 0.77,0.98) and from Black ethnic backgrounds (0.78; 0.66,0.93) less likely, to be living with obesity.

## Conclusion

Linked EHRs can provide novel insights into the shared weight status of children sharing the same household. Further qualitative research is needed to understand how household food practices may vary by other household characteristics to improve our understanding of how the home environment influences childhood obesity.

